# Quality of life in primary aldosteronism, medical vs surgical treatment: a systematic review and meta-analysis

**DOI:** 10.1007/s40618-026-02903-6

**Published:** 2026-05-06

**Authors:** Merel Wever, Christian Basile, Emanuele Bobbio, Eleftheria Gkaniatsa, Nienke Biermasz, Daniela Esposito, Oskar Ragnarsson

**Affiliations:** 1 Department of Endocrinology, Sahlgrenska University Hospital, Gothenburg, Sweden; 2 Department of Endocrinology, Leiden University Medical Center, Leiden, The Netherlands; 3 Department of Clinical Science and Education, Karolinska Institute, Stockholm, Sweden; 4 ANCMO Research Center, Heart Care Foundation, Florence, Italy; 5 Department of Cardiology, Sahlgrenska University Hospital, Gothenburg, Sweden; 6 Institute of Medicine, Sahlgrenska Academy, University of Gothenburg, Gothenburg, Sweden; 7 Wallenberg Center for Molecular and Translational Medicine, University of Gothenburg, Gothenburg, Sweden

**Keywords:** Conn’s syndrome, Spironolactone, Eplerenone, Adrenalectomy, Quality of life, Systematic review, Primary aldosteronism

## Abstract

**Introduction:**

Primary aldosteronism (PA) can be managed either by unilateral adrenalectomy (ADX) or pharmacologically with mineralocorticoid receptor antagonists (MRA). Several recent meta-analyses have examined how these treatment modalities affect cardiovascular outcomes in patients with PA. However, the impact of treatment on quality of life (QoL) remains largely unexplored.

**Objective:**

To synthesize data from previous studies that have investigated QoL in either medically or surgically treated patients with PA.

**Methods:**

A literature search was conducted in May 2025 in PubMed, Embase and Web of Science. Studies containing data on QoL before and after ADX or MRA were selected.

**Results:**

Fifteen studies evaluated QoL after treatment for PA. Most comparative studies reported greater and faster QoL improvement after ADX than with MRA. QoL consistently improved after ADX, whereas results with MRA were variable and less consistent. Patients treated with MRA were older than patients treated with ADX and frequently received low MRA doses. Five studies (259 ADX-treated and 88 MRA-treated patients) were included in a meta-analysis. Baseline QoL did not differ between treatment groups. At 6 months, QoL improved in both groups, with no statistically significant difference between ADX and MRA.

**Conclusion:**

Treatment of PA is associated with improved QoL following both ADX and MRA therapy. Although several studies suggest superior outcomes after adrenalectomy, the meta-analysis did not show a significant difference at 6 months of follow-up. The limited number of patients, short follow-up duration, and potential undertreatment with MRA represent important limitations.

**Supplementary Information:**

The online version contains supplementary material available at 10.1007/s40618-026-02903-6.

## Introduction

Primary aldosteronism (PA) is caused by excessive production of aldosterone and is the most common form of secondary hypertension [1]. The source of the increased production is either unilateral, most often due to an adrenal adenoma, or bilateral due to multiple aldosterone producing nodules [1, 2]. Unilateral PA can be treated with unilateral adrenalectomy (ADX), and bilateral PA with mineralocorticoid receptor antagonists (MRA). Without adequate treatment, PA is associated with an increased risk for cardiovascular diseases, including atrial fibrillation, stroke, myocardial infarction, heart failure, and increased mortality [3, 4].

Several recent systematic reviews and meta-analyses have examined the effects of different treatments on cardiovascular outcomes in patients with PA. The vast majority have reached the same conclusion, that ADX is superior to medical treatment [5–7]. However, none of these studies have investigated the impact on general well-being and quality of life (QoL).

A limited number of studies indicate that patients with PA may have a worse QoL compared to the general population [8, 9]. It has also been shown that both medical and surgical treatment improves QoL, where surgical treatment seems to have a greater improvement [8–10]. However, previously published studies on QoL before and after either medical or surgical treatment are few and most often based on small cohorts [11–13]. Therefore, the aim of this study was to conduct a systematic review and a meta-analysis to synthesize available evidence on the effect of treatment with either ADX or MRA on QoL in patients with PA.

## Materials and methods

This study was conducted in accordance with the Preferred Reporting Items for Systematic Reviews and Meta-Analyses (PRISMA) guidelines. The study protocol was registered in the PROSPERO database on 4 October 2022 (https://www.crd.york.ac.uk/PROSPERO/view/CRD42022362506) [14]. The study was based only on previously published data and ethical approval was therefore not required.

### Search strategy

PubMed, EMBASE, and Web of Science were searched in May 2025 for eligible articles relevant for the research question and published in January 2000 or later. The search utilized terms related to “primary aldosteronism” and “health related quality of life”. A detailed description of the search strategy is presented in the supplementary file. In addition, reference lists from relevant review articles were examined to identify any additional articles.

### Eligibility criteria

Studies assessing QoL before and after treatment with either ADX or MRA in adult patients with PA (18 years or older) were included in this systematic review.

Studies published before the year 2000, studies including < 10 patients, and papers written in a language other than English, Dutch, Italian or Swedish were excluded. If QoL data had been reported in an article, but not readily available as needed for the meta-analysis, the authors were contacted and asked to provide the raw data.

### Selection of included studies

**Fig. 1:**
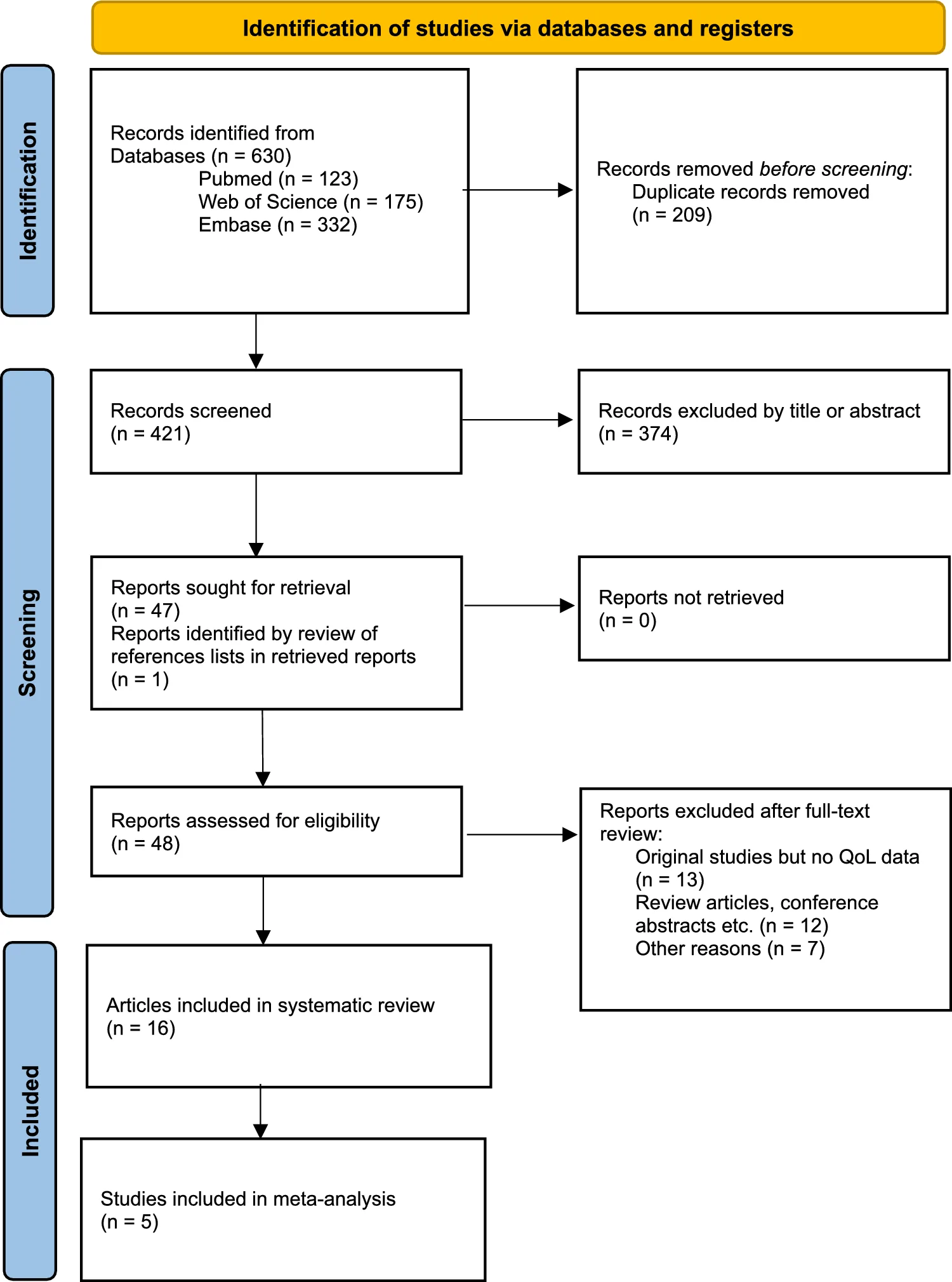
PRISMA flow chart illustrating the selection and inclusion process. PRISMA, Preferred Reporting Items for Systematic Reviews and Meta-Analyses (https://prisma-statement.org)

In total, 630 articles were identified, of which 421 remained after exclusion of duplicates (n = 209) (Fig. 1). Then, titles and abstracts (n = 421) were screened in Rayyan, an online screening program where multiple collaborators can work together, blinded from each other’s evaluation [15]. Forty-seven reports were selected and retrieved for full-text screening. One additional report was selected after reviewing reference lists from relevant review articles. Next, the full text of the selected articles (n = 48) was evaluated for eligibility and quality. All titles and abstracts, and full texts were reviewed by at least two researchers. Any conflicts were solved after a discussion with a third collaborator.

Of 48 articles, 32 were excluded; 13 did not contain any QoL data, 12 were review papers or conference abstracts, and 7 were excluded for various other reasons. Accordingly, 16 articles, from 15 studies, were included for a systematic review. For the meta-analysis, further 10 studies (11 articles) were excluded: 6 studies reporting QoL data in a form not suitable for the meta-analysis (e.g. data not reported as mean or median score, and where the authors did not reply to a request for raw data), two studies that used the Short-form 12 (SF-12), and one that used a non-validated questionnaire. Thus, 5 articles were included in the meta-analysis (Fig. 1), all using the Short Form 36 (SF-36) to evaluate QoL, an internationally validated questionnaire that has been widely used to assess QoL [16]. SF-36 contains 36 items, categorized into the following eight subdomains: physical functioning (PF), bodily pain (BP), role limitations due to physical health problems (RLPH), general health perception (GH), role limitations due to emotional health problems (RLEH), social functioning (SF), mental health (MH) and vitality (V). Scores range from 0 (worst health) to 100 (best health).

### Assessment of study quality

Quality of the studies was assessed by two researchers by using the Newcastle–Ottawa scale (Supplemental Table 1). Their evaluation was compared to making a final evaluation. A total score of 6 points or higher was considered to illustrate high quality.

**Table 1:** Summary of studies investigating QoL after treatment with MRA or adrenalectomy

Author, year [Ref] Origin	No of patients	Women, n (%)	Mean age (years)	Design, method	Main findings	MRA, sort and dose at 6 months (n)	Renin at 6 mo
Sukor^#^, 2010 [18] Australia	22 ADX	8 (36%)	50 ± 2	Prospective, SF-36	Impaired QoL at baseline that improved following ADX		6.8 ± 1.2 mU/mL Normal: 8–34 mU/mL
Ahmed^#^, 2011 [11] Australia	21 MRA	14 (67%)	55 ± 10	Prospective, SF-36	Worse QoL than the general population at baseline. Improvement at 3 and 6 months but not as great as following ADX in Sukor [17]	SPL 12.5–25 mg (n = 10) AML 2.5–10 mg (n = 9) SPL + AML (n = 2)	8.4 ± 0.3 mU/mL Normal: 8–34 mU/mL
Kunzel, 2012 [19] Germany	49 ADX, 56 MRA	50 (48%)	58 ± 13 65 ± 9	Cross-sectional, SF-12	Worse QoL during treatment with MRA for a median of 5 years compared to ADX	SPL 62 mg (15–200 mg, n = 49) EPL 57 mg (25–150 mg; n = 7)	ADX: 42 ± 128 µU/mL MRA: 86 ± 297 µU/mL Normal 8–99 µU/mL
Dekkers, 2016 [20] and Velema 2018 [10] Netherlands	92 ADX 92 MRA	26 (28%) 14 (15%)	52 ± 10 54 ± 9	Prospective, SF-36 and EQ-5D	Greater improvement 6 and 12 months following ADX than MRA	SPL 25–200 mg (n = 49) EPL max 400 mg (n = 43)	NA
Citton, 2018 [25] Italy	26 ADX	10 (38%)	54 ± 11	Prospective, SF-36	Both the physical and mental components of the SF-36 improved 6 months after ADX		NA
Ishidoya^#^, 2019 [24] Japan	179 ADX	89 (51%)	54 ± NA	Prospective, SF-36	Worse QoL at baseline compared to general population. Improved after ADX		NA
Gendreitzig, 2021 [21] Germany	93 ADX, 183 MRA	124 (41%)	52 ± 11 (all)	Prospective, SF-12	Worse mental health during treatment with MRA than after ADX (mean follow-up 2 years)	NA	NA
Buffolo^#^, 2021 [8] Italy	37 ADX 30 MRS 3 other	25 (36%)	52 ± 9 (all)	Prospective, SF-36	More pronounced improvement 6 months after ADX than MRA	SPL (n = 14) CAN (n = 16)	NA
Tan^#^, 2021 [9] Singapore	21 ADX 13 MRA	8 (38%) 2 (15%)	48 ± 11 56 ± 10	Prospective, SF-36 and EQ-5D	EQ-5D outcomes better 1 year after ADX than MRA	SPL (n = 9) EPL (n = 4)	NA
Yoshida, 2021 [27] Japan	50 MRA	29 (58%)	56 ± 13	Prospective, SF-36	Worse QoL at baseline compared to general population. Improved after MRA	SPL 29 ± 3 mg (n = 13) EPL 43 ± 2.6 (n = 30) ESAX 2.5 ± 0 (n = 7)	5.1 (3.5–10.2) pg/mL Normal: 5–45 pg/ml
Saiki^#^, 2022 [12] Japan	24 MRA	18 (75%)	60 ± NA	Prospective, SF-36	No improvement in QoL after 6 months	SPL 100 mg (n = 15) EPL 100 mg (n = 9)	2.6 (1.4–5) ng/mL/h Normal 0.5–4 ng/mL/h
Kawasaki, 2023 [26] Japan	156 ADX	80 (51%)	55 ± NA	Prospective, SF-36	Worse QoL at baseline compared to general population. Mental component of SF-36 improved after ADX		NA
Yoshida, 2024 [13] Japan	25 MRA	16 (64%)	54 ± NA	Prospective, SF-36	Improved QoL 6 months after treatment	ESAX 2.5 mg (n = 25)	4.3 (2.3–5.9) pg/mL Normal: 5–45 pg/ml
Yu, 2024 [22] China	57 ADX 59 MRA	52 (45%)	52 ± 12 (all)	Prospective, SF-36	Greater improvement in QoL 12 months after ADX than MRA	MRA (n = 59)	NA
Ananda, 2024 [23] International	246 ADX 442 medical treatment	455 (67%)	44 ± 13 (all)	Cross-sectional on-line survey. 5-point Likert scale: “I have noticed an improvement in my QoL after receiving treatment for PA”	Patients treated with ADX more likely to experience improved QoL (odds ratio, 2.36 [95% CI, 1.67–3.35]) compared to medically treated patients	NA	NA

ADX, adrenalectomy; AML, amiloride; CAN, canrenoate, EPL, eplerenone; ESAX, esaxerenone; EQ-5D, EuroQol Five Dimensions; MRA, mineralocorticoid receptor antagonist; PA, primary aldosteronism; SF, short form, SPL, spironolactone; QoL, quality of life

^#^Studies included in the meta-analysis

### Data synthesis and statistical analysis

The QoL data for the meta-analysis was extracted and collected in excel and then transferred to R (version 4.5.0, meta package). The data that was collected were mean QoL scores together with standard deviations. If data was reported as median with quartiles it was converted to mean and standard deviation using the methods described by Wan et al. [17]. Study and patient characteristics such as age, gender distribution, follow-up time, type of treatment (unilateral adrenalectomy or mineralocorticoid receptor antagonists), lateralization of PA (unilateral, bilateral or unknown), adrenal venous sampling (AVS), plasma renin following treatment, and type and doses of MRA were also collected.

After data extraction, a random effects model was employed to compare the QoL scores using the inverse-variance method to account for expected between-study heterogeneity. The results are displayed in forest plots with 95% confidence intervals. A p-value of < 0.05 was considered to be statistically significant. Additionally, the Higgins statistic I^2^ and tau2 were calculated. The tau2 was estimated using the Restricted Maximum Likelihood (REML) estimator. The heterogeneity testing, I^2^, shows acceptable (0—40%), moderate (30—60%), substantial (50—90%) and strong (75—100%) heterogeneity according to the Cochrane Handbook for Systematic Reviews.

## Results

### Systematic review

Sixteen articles from 15 observational studies containing QoL data after treatment were identified (Table 1). No randomized controlled trial was identified. The mean age of the patient cohorts treated with ADX ranged between 48 and 58 years, and between 54 and 65 years in cohorts of patients treated with MRA. Eight of 15 studies assessed and compared QoL in patients treated with ADX and patients receiving MRA (Table 1). In the first two studies, coming from the same center, including 22 patients treated with ADX and 21 patients treated with MRA, QoL improved after both treatments, but more slowly and to a lesser extent after MRA than patients treated with ADX [11, 18]. Similarly, in a cross-sectional study, QoL was worse in patients who had been treated with MRA for a median of 5 years compared to patients who had been operated 4 years earlier [19]. Furthermore, in the SPARTACUS trial, both physical and mental component summary score of the SF-36 questionnaire improved after ADX but not MRA, and greater beneficial effects on seven of eight SF-36 subscales, and the VAS scores on the EQ-5D, were seen 6 and 12 months after ADX compared to MRAs [10, 20]. Further studies have shown worse score for mental health during treatment with MRA than after ADX [21], and greater improvement in QoL after ADX than MRA [8, 9, 22]. Finally, in a large international survey, answered by 684 patients with PA, of whom 246 were treated with ADX, showed that operated patients were more likely to experience improved QoL compared to medically treated patients [23].

Four studies investigated QoL before and after ADX [18, 24–26]. All these studies showed impaired QoL before ADX and a significant improvement postoperatively. Three studies investigated QoL before and after treatment with MRA. In one of these, QoL was worse at baseline in patients compared to normative data, improved three and six months after treatment with MRA (mainly eplerenone) but did not normalize [27]. The doses of MRA were in general low as was active renin concentrations or plasma renin activity (Table 1). In another study, no difference in QoL was seen at baseline compared to the general population [13]. Nevertheless, after treatment with esaxerenone, a significantly improved QoL was observed. Finally, in a study using either spironolactone or eplerenone, no improvements in QoL was observed after 6 months of treatment [12].

### Meta-analysis

Five articles were included in the meta-analysis (Table 1). The articles were published between 2011 to 2022. Three studies investigated QoL both in ADX and MRA treated patients [8, 9, 11], one study in only MRA treated patients [12] and one in only patients treated with ADX [24]. Four of the five studies reported QoL at baseline and at 6 months of follow-up. One study reported QoL at baseline and 6 to 12 months of follow-up [9]. In total, 259 patients were treated with ADX and 88 with MRA. The mean age of the cohorts ranged from 48 to 54 years in patients treated with ADX and from 55 to 60 years in patients treated with MRA. The proportion of women varied between 8 to 51% in the ADX cohorts and between 15 to 75% in the MRA cohorts across studies. All studies specified the type of MRA used and the number of patients treated with each agent (Table 1). Overall, spironolactone was the most frequently used MRA, followed by eplerenone and canrenoate. Two of the four studies reported the administered daily MRA doses: one study used spironolactone 12.5–25 mg (or amiloride 2.5–10 mg) [11], while the other administered spironolactone 100 mg or eplerenone 100 mg [12].

**Fig. 2:**
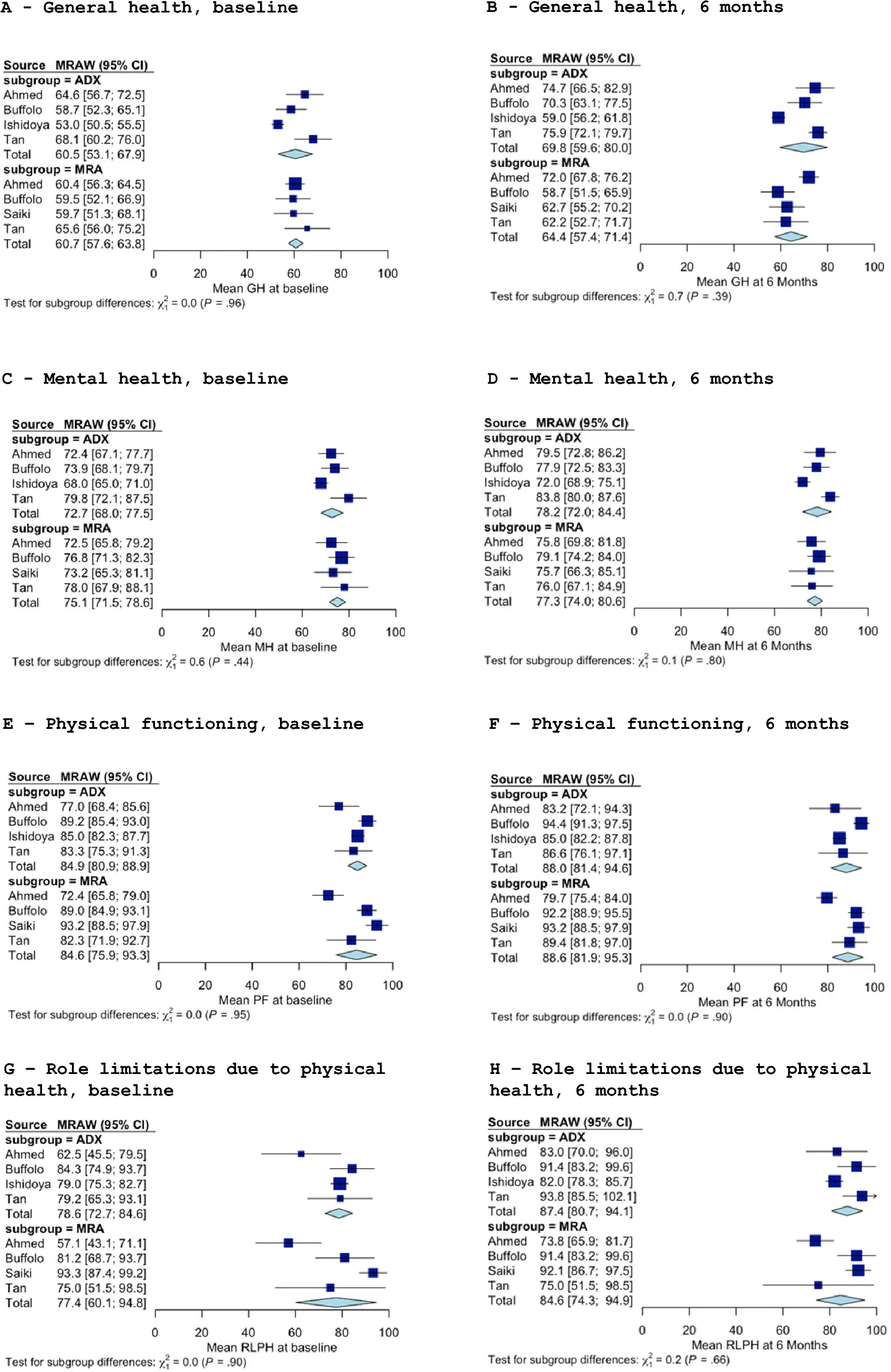

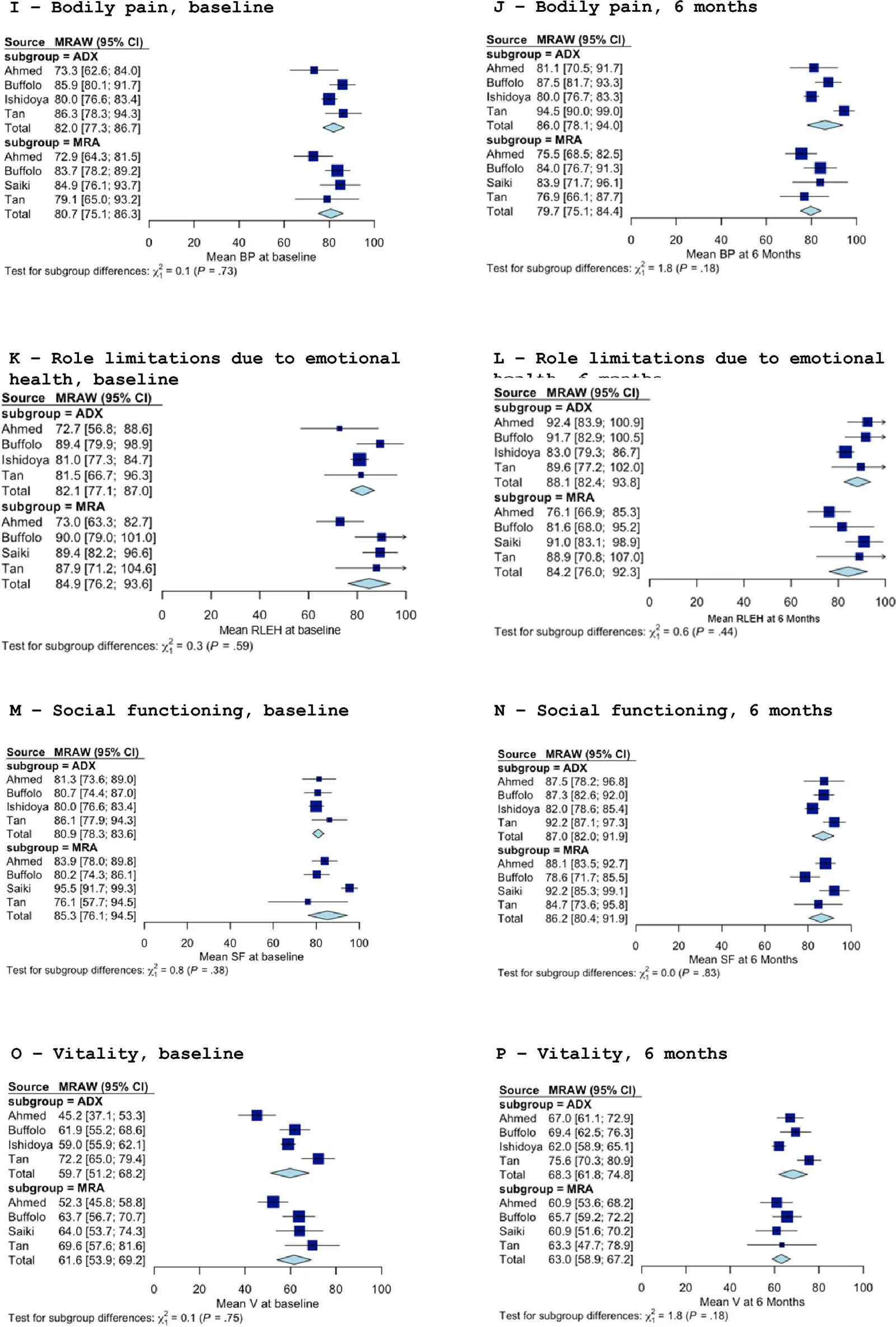
Forest plots showing the mean scores for the eight subdomains of quality of life, evaluated with SF-36, before and 6 months following adrenalectomy or from start with mineralocorticoid receptor antagonist; (**A, B**) general health, (**C, D**) mental health, (**E, F**) physical functioning, (**G, H**) role limitations due to physical health problems, (**I, J**) bodily pain, (**K, L**) role limitations due to emotional health problems, (**M, N**) social functioning, (**O, P**) vitality

At baseline (e.g. before disease-specific treatment), there were no significant differences in mean scores across any of the QoL subdomains between patients treated with ADX and those treated with MRA (Fig. 2). After 6 months, QoL improved but no difference was observed between the groups (Fig. 2). Similarly, analysing change in scores (ΔQoL from baseline to follow-up) did not reveal any difference between the groups (Supplementary figure S1).

Four studies [8, 9, 11, 12] included in the meta-analysis were considered to be of high quality and one study [24] of moderate (Supplementary figure S1). Heterogeneity testing, both for baseline and 6 months, mostly showed moderate to substantial heterogeneity (I^2^ 40—80%; Supplemental table S2).

## Discussion

In this systematic review and meta-analysis, we evaluated the impact of ADX and MRA therapy on QoL in patients with PA. Across most individual studies, ADX was associated with greater and more rapid improvements in QoL, particularly in mental health domains, compared with medical therapy. However, despite these consistent trends, our meta-analysis showed no significant difference in overall QoL between treatment strategies at 6 months follow-up, with both approaches resulting in meaningful improvement. These findings highlight the complexity of comparing surgical and medical treatment effects on patient-reported outcomes and underscore the need for longer-term and more standardized assessments of QoL in this population. The included studies varied in design, age and sex distribution, and type and dosage of MRA used. Thus, given the limited number of studies, differences in methodological quality and significant heterogeneity, the results should be interpreted with caution.

There may be genuine differences in QoL outcomes between treatment modalities. MRA therapy is associated with a higher medication burden compared with surgical treatment, which may negatively influence patients’ perception of their health status [28]. The need for daily medication can serve as a constant reminder of illness, in contrast to the sense of recovery often experienced after curative surgery, achieved in more than 85% of all operated patients [28–31]. In addition, adverse effects, particularly those related to spironolactone, may further contribute to reduced QoL among patients receiving MRA therapy. Nevertheless, baseline differences between treatment groups complicate direct comparisons and represent a potential source of residual confounding. Patients selected for ADX are typically younger and have fewer comorbidities, whereas those treated with MRA are often older and have a higher burden of comorbidity. These differences may influence both baseline QoL and the magnitude of improvement following treatment. As most included studies were observational, such confounding cannot be fully accounted for and should be considered when interpreting the results. Age and comorbidities, especially, are important factors that have a negative impact on QoL scores [30]. Recent data, however, suggest that reduced QoL in PA may not be specific to aldosterone excess [32]. A large cohort study comparing patients with PA and primary hypertension reported no significant differences in QoL between the groups at baseline, suggesting that impaired QoL may be more closely related to blood pressure control and treatment burden than to aldosterone excess per se.

Undertreatment of patients receiving MRA contributes to worse cardiovascular outcome compared to patients receiving adequate MRA doses [3, 4]. Unfortunately, undertreatment is common. In a recent large multicenter study, approximately 50% of patients on long-term medical treatment for PA where not receiving adequate doses, and higher doses were associated with better outcome [33]. The doses used in the studies that were included in current analysis, when provided, were generally low in comparison to recommendations in international guidelines [34]. The mean doses of spironolactone in two of the studies for instance, were 12.5 – 25 mg [11] and 29 mg [27], respectively, which is much lower than the recommended dose. Thus, undertreatment may also be an important contributor to poor QoL in medically treated patients, and the influence of increasing MRA doses to optimal levels on QoL outcomes should be investigated in future studies.

Several limitations of the meta-analysis should be acknowledged. Substantial heterogeneity was observed within both treatment groups, warranting cautious interpretation of the results. Moreover, the selected 6-month follow-up period may be too short to capture meaningful changes in QoL, especially in patients receiving medical treatment as the up titration of the MRA may take longer time; follow-up intervals of 12 months or more might provide a more accurate assessment of long-term improvement. Although some studies have reported data beyond 6 months [9, 24], their number was insufficient for separate analysis. Additional subgroup analyses based on factors such as nationality, medication type and dosage, or sex could have further clarified between-group differences. Furthermore, information on cortisol and aldosterone co-secretion was not consistently reported across studies and could therefore not be evaluated, although this may influence QoL outcomes. Beyond general QoL, other psychological dimensions may also be affected in patients with PA but were not included in the current study. Previous studies have suggested increased prevalence of anxiety and depressive symptoms in this population, with some improvement following treatment [9, 32, 35]. Finally, the small overall sample size primarily reflects the general lack of available data on QoL in patients with PA, highlighting the need for future research focused on patient-reported outcomes alongside therapeutic efficacy.

In conclusion, both ADX and MRA therapy are associated with improvements in QoL in patients with PA. While individual studies suggest greater and more rapid benefits after ADX, short-term meta-analytic data do not demonstrate a clear difference between treatment modalities, potentially reflecting heterogeneity, baseline differences, and suboptimal medical therapy. Future well-designed studies with long-term follow-up are needed, including systematic and standardized assessment of QoL as an important but under-evaluated patient-reported outcome. Such studies should incorporate correlations with clinical outcomes, including PASO [36] and PAMO [33] criteria, and consider sex-specific differences to better define treatment-specific effects and guide individualized management strategies.

## Supplementary Information

Below is the link to the electronic supplementary material.

Supplementary file1

## Data Availability

A protocol of this study is available at the PROSPERO website (CRD42022362506) (https://www.crd.york.ac.uk/PROSPERO/view/CRD42022362506). All aggregate patient data are presented in the manuscript or the appendix. Individual patient data cannot be made publicly available due to the character of the study.
