# Supplementary material for: Quality of life in primary aldosteronism, medical vs surgical treatment: a systematic review and meta-analysis
Source: J Endocrinol Invest. 2026 May 6;49(9):2305–13. doi: 10.1007/s40618-026-02903-6 (PMC13498613; doi:10.1007/s40618-026-02903-6)
Supplement: Supplementary file 1 [file 40618_2026_2903_MOESM1_ESM.docx]

**Supplementary appendix**

**Quality of life in primary aldosteronism, medical vs surgical treatment – a systematic review and meta-analysis**

**Authors:** Merel Wever, Christian Basile, Emanuele Bobbio, Eleftheria Gkaniatsa, Nienke Biermasz, Daniela Esposito, and Oskar Ragnarsson

**Content**

Page 2 – A detailed description of the search strategy

Page 4 – Supplementary figure 1: change in QoL scores from baseline to follow-up

Page 4 – Supplementary table 1: Quality assessment of the studies included in the meta-analysis according to the Newcastle-Ottawa scale

Page 5 – Supplementary table 2: Heterogeneity testing for Heterogeneity testing for baseline and 6 months for the studies included in the meta-analysis

**A detailed description of the search strategy**

PubMed

| Interface: pubmed.gov  Date of Search: 2025-05-19  Number of hits: 123  Comment: | Field labels   - [mh] or [mesh] = exploded MeSH term - [tiab] = title, abstract - [tw] = textwords: all words and numbers in the title, abstract, other abstract, MeSH terms, MeSH Subheadings, Publication Types, Substance Names * = truncation of word for alternate endings NB If truncating a phrase – the truncated term must be the last word in the phrase. |
| --- | --- |
| **#** \| **Searches** \| **Results**; 1 \| ("Hyperaldosteronism"[Mesh] OR "Hyperaldosteronism"[tiab] OR "Aldosteronism"[tiab] OR "Conn Syndrome"[tiab] OR "Conn’s Syndrome"[tiab] OR "Conns Syndrome"[tiab]) \| 13,047; 2 \| ((((EQ5D[tiab] OR EQ-5D[tiab] OR EQ 5D[tiab] OR EuroQoL[tiab] OR EQ-5D-L3[tiab] OR EQ-5D-L5[tiab] OR EQ5D-L5[tiab]) OR ((Anxiety[mesh] OR Anxiety[tiab] OR Angst[tiab] OR Nervousness[tiab] OR Hypervigilance[tiab] OR anxiousness[tiab] OR Anxiety Disorders[mesh]))) OR ((Quality of life[mesh] OR Quality of life[tiab] OR Life quality[tiab] OR HqL[tiab] OR hqol[tiab] OR hrqol[tiab] OR QoL[tiab] OR personal satisfaction[mesh] OR personal satisfaction*[tiab] OR life satisfaction*[tiab]))) OR ((depression[mesh] OR depression[tiab] OR Depressive[tiab] OR depressive disorder[mesh] OR melancholia*[tiab]))) OR ("SF-36"[tiab] OR "SF-12"[tiab] OR "SF 36"[tiab] OR "SF 12"[tiab] OR Short form 12[tiab] OR Short form-12[tiab] OR Short form 36[tiab] OR Short form-36[tiab] OR Short form health survey 12[tiab] OR Short form health survey-12[tiab] OR Short form health survey 36[tiab] OR Short form health survey-36[tiab]) \| 1,275,636; 3 \| #1 AND #2 \| 151; 4 \| Limit to publication year 2000-2026 \| 123 | |

Web of Science Core Collection

| Interface: Clarivate Analytics  Date of Search: 2025-05-19  Number of hits:175 | Field labels   - TS/Topic = title, abstract, author keywords and Keywords Plus - NEAR/x = within x words, regardless of order - * = truncation of word for alternate endings |
| --- | --- |
| **#** \| **Searches** \| **Results**; 1 \| TS=( hyperaldosteronism OR hyperaldosteronemia OR aldosteronism OR aldosteronemia OR Hyperaldosteronism OR "Conn Syndrome" OR "Conn's Syndrome" OR "Conns Syndrome") \| 10,573; 2 \| TS=(("EQ5D" OR "EQ-5D" OR "EQ 5D" OR "EuroQoL" OR "EQ-5D-L3" OR "EQ-5D-L5" OR "EQ5D-L5") OR ("Anxiety" OR "Angst" OR "Nervousness" OR "Hypervigilance" OR "Anxiousness" OR "Anxiety Disorders") OR ("Quality of life" OR "Life quality" OR "HqL" OR "hqol" OR "hrqol" OR "QoL" OR "Personal satisfaction" OR "life satisfaction") OR ("Depression" OR "Depressive" OR "Depressive disorder" OR "Melancholia") OR ("SF-36" OR "SF-12" OR "SF 36" OR "SF 12" OR "Short form 12" OR "Short form-12" OR "Short form 36" OR "Short form-36" OR "Short form health survey 12" OR "Short form health survey-12" OR "Short form health survey 36" OR "Short form health survey-36")) \| 1,634,510; 3 \| #1 AND #2 \| 189; 4 \| #3 AND Publication Date 2000-01-01 – 2026-01-01 \| 175 | |

Embase

| Interface: Elsevier  Date of Search: 2025-05-19  Number of hits: 332  Comment: use ' for phrase searching | Field labels   - /exp = exploded Emtree term - /de = non exploded Emtree term - :ti,ab,kw. = title, abstract and author keywords - NEAR/n = within x words, regardless of order - * = truncation of word for alternate endings - * = variable wildcard, i.e one or more letters - ' = phrase |
| --- | --- |
| **#** \| **Searches** \| **Results**; 1 \| hyperaldosteronism:ti,ab OR hyperaldosteronemia:ti,ab OR aldosteronism:ti,ab OR aldosteronemia:ti,ab OR 'hyperaldosteronism'/exp OR hyperaldosteronism OR 'conn syndrome':ti,ab OR 'conns syndrome':ti,ab OR 'syndrome, conn*':ti,ab \| 20,731; 2 \| ('EQ5D':ti,ab,kw OR 'EQ-5D':ti,ab,kw OR 'EQ 5D':ti,ab,kw OR 'EuroQoL':ti,ab,kw OR 'EQ-5D-L3':ti,ab,kw OR 'EQ-5D-L5':ti,ab,kw OR 'EQ5D-L5':ti,ab,kw) OR ('anxiety'/exp OR 'anxiety':ti,ab,kw OR 'angst':ti,ab,kw OR 'nervousness':ti,ab,kw OR 'hypervigilance':ti,ab,kw OR 'anxiousness':ti,ab,kw OR 'anxiety disorder'/exp) OR ('quality of life'/exp OR 'quality of life':ti,ab,kw OR 'life quality':ti,ab,kw OR 'HqL':ti,ab,kw OR 'hqol':ti,ab,kw OR 'hrqol':ti,ab,kw OR 'QoL':ti,ab,kw OR 'personal satisfaction'/exp OR 'personal satisfaction*':ti,ab,kw OR 'life satisfaction*':ti,ab,kw) OR ('depression'/exp OR 'depression':ti,ab,kw OR 'depressive':ti,ab,kw OR 'depressive disorder'/exp OR 'melancholia*':ti,ab,kw) OR ('SF-36':ti,ab,kw OR 'SF-12':ti,ab,kw OR 'SF 36':ti,ab,kw OR 'SF 12':ti,ab,kw OR 'Short form 12':ti,ab,kw OR 'Short form-12':ti,ab,kw OR 'Short form 36':ti,ab,kw OR 'Short form-36':ti,ab,kw OR 'Short form health survey 12':ti,ab,kw OR 'Short form health survey-12':ti,ab,kw OR 'Short form health survey 36':ti,ab,kw OR 'Short form health survey-36':ti,ab,kw) \| 2,531,492; 3 \| #1 AND #2 \| 575; 4 \| #3 AND ('article'/it OR 'article in press'/it OR 'review'/it) \| 363; 5 \| #4 AND (2000:py OR 2001:py OR 2003:py OR 2004:py OR 2005:py OR 2006:py OR 2007:py OR 2008:py OR 2009:py OR 2010:py OR 2011:py OR 2012:py OR 2013:py OR 2014:py OR 2015:py OR 2016:py OR 2017:py OR 2018:py OR 2019:py OR 2020:py OR 2021:py OR 2022:py OR 2023:py OR 2024:py OR 2025:py) \| 332 | |

**Supplementary figure 1**

**Supplementary table 1: Quality assessment of the studies included in the meta-analysis according to the Newcastle-Ottawa scale**

| Section | Selection | | | | Comparability^1^ | Outcome | | | Total stars |
| --- | --- | --- | --- | --- | --- | --- | --- | --- | --- |
| Author, year | Representativeness of exposed cohort | Selection of the (non)-exposed cohort | Ascertainment of exposure | Demonstration that outcome of interest was  not present at start of study | Comparability of cohorts on the basis of the design or analysis | Assessment of outcome | Was follow-up long enough for outcomes to occur | Adequacy of follow-up of cohorts |  |
| Ahmed, 2011 | * | * | * | * | * | - | * | - | 6 |
| Buffolo, 2020 | * | * | * | * | ** | - | * | * | 8 |
| Ishidoya, 2019 | * | - | * | * | - | - | * | - | 4 |
| Saiki, 2022 | - | * | * | * | * | - | * | * | 7 |
| Tan, 2021 | * | * | * | * | * | - | * | - | 7 |

^1^Comparability: 1 star is awarded for controlling of age and gender. An additional star is given for controlling of additional factors such as intensity of antihypertensive treatment or presence of comorbidity.

**Supplementary table 2: Heterogeneity testing for baseline and 6 months for the studies included in the meta-analysis**

**Baseline**

|  | PF | RLPH | BP | GH | RLEH | SF | MH | V |
| --- | --- | --- | --- | --- | --- | --- | --- | --- |
| Tau | 5.0 | 9.0 | 2.8 | 4.9 | 4.4 | 43.5 | 3.2 | 6.6 |
| Tau2 | 25.3 | 81.6 | 8.0 | 23.9 | 19.0 | 6.6 | 10.6 | 43.4 |
| I2 | 80.3% | 79.7% | 40.6% | 74.5% | 48.2% | 83.8% | 55.6% | 79.4% |

**6 months**

|  | PF | RLPH | BP | GH | RLEH | SF | MH | V |
| --- | --- | --- | --- | --- | --- | --- | --- | --- |
| Tau | 5.2 | 6.1 | 6.4 | 7.8 | 3.9 | 3.8 | 4.1 | 4.7 |
| Tau2 | 27.1 | 37.8 | 41.0 | 61.6 | 15.6 | 14.5 | 16.7 | 21.7 |
| I2 | 84.3% | 73.3% | 80.2% | 89.6% | 46.0% | 64.3% | 70.4% | 69.3% |
